# High strain-level diversity of *Bradyrhizobium* across Australian soils

**DOI:** 10.1093/ismejo/wraf222

**Published:** 2025-10-08

**Authors:** Clifton P Bueno de Mesquita, Matthew R Olm, Andrew Bissett, Noah Fierer

**Affiliations:** Cooperative Institute for Research in Environmental Sciences, University of Colorado, 1665 Central Campus Mall, Boulder, CO 80309-0216, United States; Department of Ecology and Evolutionary Biology, University of Colorado, 1900 Pleasant St., Boulder, CO 80309-0334, United States; Department of Integrative Physiology, University of Colorado, 1900 Pleasant St., Boulder, CO 80309-0354, United States; CSIRO, Oceans and Atmosphere, Hobart, Tasmania 7004, Australia; Cooperative Institute for Research in Environmental Sciences, University of Colorado, 1665 Central Campus Mall, Boulder, CO 80309-0216, United States; Department of Ecology and Evolutionary Biology, University of Colorado, 1900 Pleasant St., Boulder, CO 80309-0334, United States

**Keywords:** biogeography, strains, ubiquity, pangenome, comparative genomics, metagenomics, *Bradyrhizobium*

## Abstract

Global surveys of soil bacteria have identified several taxa that are nearly ubiquitous and often the most abundant members of soil bacterial communities. However, it remains unclear why these taxa are so abundant and prevalent across a wide range of soil types and environmental conditions. Here, we use genome-resolved metagenomics to test the hypothesis that strain-level differences exist in these taxa that are not adequately captured with standard marker gene sequencing, and that distinct strains harbor unique traits that reflect adaptations to different soil environments. We analyzed data from 331 natural soils spanning Australia to assess strain differentiation in *Bradyrhizobium*, a dominant soil bacterial genus of ecological importance. We developed a workflow for strain-level bacterial analyses of complex soil metagenomes, combining genomes from pre-existing databases with new genomes generated via targeted assembly from metagenomes to detect 181 *Bradyrhizobium* strains across the soil collection. In addition to a high degree of phylogenetic variation, we observed substantial variation in pangenome content and inferred traits, highlighting the breadth of diversity within this widespread genus. Although members of the genus *Bradyrhizobium* were detected in >80% of samples, most individual strains were restricted in their distributions. The overall strain-level community composition of *Bradyrhizobium* varied significantly across geographic space and environmental gradients, and was particularly associated with differences in temperature, soil pH, and soil nitrate and metal concentrations. Our work provides a general framework for studying the strain-level ecology of soil bacteria and highlights the ecological and pangenomic diversity within this dominant soil bacterial genus.

## Introduction

Soils harbor some of the most diverse bacterial communities on Earth [1]. As in nearly all biological communities, most soil bacterial taxa are rare and only a few are abundant in any particular sample [2–4]. Moreover, the bacterial taxa that are abundant in any individual soil often tend to be highly abundant and highly prevalent across many different soils that span a range in environmental conditions [2, 5–7]. This observation then raises the question of why certain bacterial taxa are so abundant and prevalent in soil. How are these “dominant” taxa, defined as in Delgado-Baquerizo et al. (2018) [2], able to thrive in vastly different soil environments? The most parsimonious explanation is there are species- to subspecies-level differences within a given taxon across different environments, with high strain-level diversity corresponding to high trait diversity, such that members of a single taxon can persist across a range of environmental conditions [8]. In other words, a bacterial genus or species that appears to be dominant across many soils is actually composed of many distinct strains with distinct niches. Here we define a strain as a taxon with a unique genome, with strain-level diversity describing diversity at an even finer level of resolution than species.

There is a growing body of research in both environmental and host-associated systems highlighting the value of strain-level analyses for exploring the structure of bacterial communities and identifying spatiotemporal patterns that may not be apparent at coarser levels of taxonomic resolution [9]. For example, Cho and Tiedje (2000) observed endemism of different *Pseudomonas* species in different locations, suggesting that the species within the genus were not globally mixed [10]. More recently, Coleman and Chisholm (2010) found that there was a large fraction of genes that were rare in populations of two abundant marine bacteria, reflecting continual gene transfer and loss [11]. Furthermore, a pangenomic study of *Prochlorococcus,* one of the most highly prevalent marine bacterial genera, revealed that subtle differences in gene cluster content were associated with biogeographic patterns [12]. Even in the human microbiome, recent work has shown how strain-level analyses can be used to understand bacterial community assembly in infant guts [13, 14].

**Figure 1:**
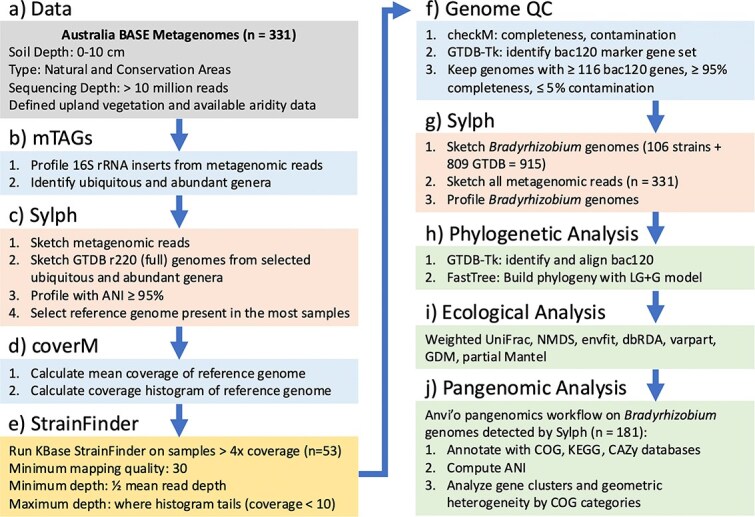
Methodological flow diagram including the starting data, identification of highly prevalent and abundant genera, selection of reference genomes, coverage calculation, targeted genome assembly with StrainFinder, genome QC, genome detection and relative abundance calculation (profile) with Sylph, and downstream phylogenetic, ecological, and pangenomic analyses.

Shotgun metagenomic sequencing and subsequent genome-resolved analyses can help refine taxonomic identifications and functional gene analyses to the species and strain levels of resolution [15]. There are many tools for such strain-based analyses of bacteria using metagenomic data, including reference-based and de novo approaches, which have been reviewed previously [16–20]. Such tools are frequently used to investigate the human gut microbiome where the reference genome databases are reasonably comprehensive [18–23]. Performing strain-level analyses on environmental samples, particularly soils, is far more challenging due to the complexity of these communities and the paucity of reference genomes available for many members of these communities, even the more abundant taxa [24]. In recent years, reference genome databases have grown exponentially in size and the greater sequencing depth that can be achieved with newer sequencing technologies has enabled more effective analyses of complex environmental microbiomes. Still, given the reference genome database limitations, it is important to supplement those databases with genomes assembled directly from metagenomic datasets of interest. Although strain-level analyses of such de novo metagenome-assembled genomes (MAGs) have been the focus of previous work in soils and seawater [25, 26], here we develop a general methodological framework that combines available reference genome data with targeted assembly of additional genomes directly from soil metagenomes (Fig. 1). Whereas much previous strain-based research has focused on population genomics [21, 26, 27], we combine strain detection with compositional tools to conduct ecological and pangenomic analyses on detected strains.

We used the approach of combining reference genomes with targeted MAG assembly to investigate the strain-level diversity of the dominant soil bacterial genus *Bradyrhizobium* using a standardized collection of soil metagenomes from across Australia. *Bradyrhizobium* has consistently been found to be one of the most prevalent and abundant members of soil bacterial communities in large-scale 16S rRNA gene sequencing efforts [2, 5–7, 28]. For example, in an analysis of 16S rRNA gene data from 275 Australian soils, four of the top five most abundant amplicon sequence variants (ASVs) were members of the *Bradyrhizobium* genus; these ASVs were also among the most prevalent ASVs, being detected in >74% of samples [6]. *Bradyrhizobium* are most well known as taxa that symbiotically fix nitrogen (N) with various plant hosts in the legume family and thus play an important role in N cycling and ecosystem productivity [29, 30]. *Bradyrhizobium* spp. containing a genomic island of *nif* genes for nitrogen fixation were found to be widespread in a variety of environments [31]. Indeed, *Bradyrhizobium* spp. are the dominant diazotrophs in many soils, often comprising over 30% of the N-fixing soil bacterial community [32–34]. However, the genus *Bradyrhizobium* also contains free-living soil-inhabiting taxa and taxa capable of photosynthesis [35–37]. While long known as symbionts of both legumes and non-legumes [29, 30], more recent work has highlighted the importance of non-symbiotic *Bradyrhizobium* in soils, as non-symbiotic members of the genus were found to dominate North American forest soils [37]. Given the dominance of the *Bradyrhizobium* in many soil bacterial communities and given the genomic and ecological diversity represented within this genus, we expected that we could use our approach (Fig. 1) to conduct genome-resolved analyses of this genus and provide insights into the biogeographical patterns exhibited by this taxon that could not be resolved using more standard marker gene sequencing approaches.

We conducted strain-level genomic analyses of species within the genus *Bradyrhizobium* to investigate the biogeography and pangenome of this genus directly from 331 shotgun metagenomes representing a broad range of soil types from across the continent of Australia. We used the resulting data from our approach to test three hypotheses. First, we hypothesized that no single *Bradyrhizobium* strain is dominant in soils across a wide range of sites and environmental conditions, but rather there is extensive strain-level diversity within the genus, with specific strains having more restricted distributions. Second, we hypothesized that geographic distance and environmental differences in both soil properties and climate variables are associated with *Bradyrhizobium* strain-level community composition, and this drives the restricted distributions of the strains. Finally, we hypothesized that the detected *Bradyrhizobium* strains collectively have a large pangenome that encompasses varying life history and metabolic strategies among strains and reflects extensive ecological diversification within the genus.

## Materials and methods

### Data

Quality-filtered shotgun metagenomic data (150 bp reads) were downloaded from the Australian BASE program via a request in their online web portal [38]. We selected 331 soil samples that met the following criteria: 0–10 cm depth, from natural areas (designated “natural and conservation areas” in BASE), defined upland vegetation types, sequencing depth of at least 10 million reads, and available data on climate characteristics (see below) (Fig. 1). These samples were collected from throughout the year (Table S1). We downloaded all associated sample and environmental metadata from BASE, which included edaphic properties such as organic carbon content, nitrate concentration, pH, available phosphorus, and other biogeochemical variables [38] (Fig. S1, Table S1). To complement the soil data, we acquired climate data from publicly available sources. Aridity index was determined for each sample using the Global Aridity Database version 3 map at 30 arc-second resolution [39], and mean annual temperature and precipitation were determined using the WorldClim2 database [40]. The 331 selected samples were distributed across most of the Australian mainland and the island of Tasmania and spanned large gradients in both climate (including arid, semi-arid, dry sub-humid, and humid climate classifications, [39]) and soil properties (Fig. S2).

### Genome selection and targeted assembly

To confirm the abundance and ubiquity of *Bradyrhizobium*, we first selected a subset of 104 samples spanning most of the aridity gradient, focusing on a subset of samples initially to save computational resources. We profiled the raw metagenomic reads taxonomically using mTAGs [41], which identifies 16S and 18S rRNA gene sequences in the metagenomes and assigns taxonomy by aligning them to the SILVA v138.1 database [42] with VSEARCH [43] (Fig. 1). We also ran mTAGs on the remaining samples to assess *Bradyrhizobium* relative abundance across all samples. We then downloaded all of the genomes of highly prevalent (75% of samples) and abundant (mean > 0.1% relative abundance across the whole dataset) genera from the full version of GTDB r220 [44] by first downloading the GTDB metadata to get the NCBI genome accessions for those genera, and then using the NCBI datasets toolkit [45] to download the genomes (n = 21 467) by accession. Next, to identify the best reference genomes for read mapping, we used Sylph [46] to build sketches of the 104 metagenomes and 21 467 genomes, and determine which genomes were present using “profile” mode and a 95% containment average nucleotide identity (ANI) cutoff (Fig. 1). To calculate read coverage and relative abundance, we then used coverM [47] to map reads to the most prevalent genomes (Fig. 1). A *Bradyrhizobium* genome (*Bradyrhizobium diazoefficiens_F*, GCA_016616885.1) was detected with mean coverage >4x in the most samples (n = 53 of 104). High coverage is crucial for confident single nucleotide variant (SNV) calling and strain identification. We ran coverM with “method = mean” to calculate mean coverage and “method = coverage_histogram” to generate the histogram of coverage per base. Both methods were used to inform parameters in StrainFinder (see below) (Fig. 1).

The goal of using StrainFinder was to perform targeted assembly of *Bradyrhizobium* MAGs to capture diversity that is not captured in pre-existing reference genome databases. Although *Bradyrhizobium* genomes are reasonably well-represented in reference databases (869 genomes in GTDB r220), we were concerned that the pre-existing genomes may not necessarily represent the full extent of *Bradyrhizobium* diversity in soil, especially because most (~90%) of the pre-existing *Bradyrhizobium* genomes in GTDB are derived from cultured isolates and many members of this genus have not yet been cultivated [48]. Strains were assembled with the KBase [49] implementation of StrainFinder [23], which maps reads to reference genomes, calls SNVs, and identifies strains based on SNV frequencies. We only profiled samples with mean coverage >4x and at least 40% of the genome covered (n = 53). The KBase implementation then performs the additional steps of remapping reads to the consensus strains and assembling complete genomes for each strain. We used a default value of 30 for the read mapping quality parameter. We used the mean coverage output from coverM for each sample to define the minimum depth parameter for SNV calling in StrainFinder, which was set to half of the mean read depth. To avoid determining SNVs in regions that represent repeats, we set the maximum depth parameter to before the tail end of the coverage histogram for each sample. We defined the beginning of the “tail” of the coverage histogram as the coverage value at which the number of bases with that value was below 10. By default, four strains per sample were assembled. We then used FastANI [50] to calculate the ANI among the strains and between the strains and the reference genome. Completeness and contamination of each strain were calculated with checkM [51].

To build a *Bradyrhizobium* genome database, we combined the 106 MAGs from StrainFinder (the top two strains per sample) with the 869 *Bradyrhizobium* genomes in GTDB r220, for a total of 975 *Bradyrhizobium* genomes. We used GTDB-tk [52] to identify a set of 120 single copy marker genes from those genomes (bac120). We filtered out any genome that was missing more than 4 of the bac120 genes, which yielded 915 genomes. The StrainFinder MAGs all had completeness >95% and contamination <1% and the GTDB genomes all had completeness >95% and contamination <5% (Fig. 1). These 915 genomes also included 19 commercial *Bradyrhizobium* inoculants used in Australian agriculture [53]. We used Sylph to sketch these 915 genomes and create a strain-by-sample relative abundance matrix across all 331 metagenomes using profile mode and a containment ANI detection cutoff of 95% (Fig. 1). Our approach of first generating new strains and then generating a strain-level abundance profile is similar conceptually to that of StrainScan [54]; we opted to use StrainFinder and Sylph because of the accuracy of the SNV-based maximum likelihood method of StrainFinder and the speed and accuracy of the containment ANI-based method of Sylph.

### Phylogeny and pangenomics

To build a phylogenetic tree of the starting 915 genomes and the Sylph-detected genomes (n = 181, see Results), we used GTDB-tk to align the previously identified bac120 genes. We used *Nitrobacter winogradskyi* Nb-255 (GCF_000012725.1) [55] as an outgroup to root the tree. Instead of randomly selecting 42 amino acids per gene (default setting), we initially included all amino acids per gene, but to minimize the effects of gaps, amino acids were retained only if they were present in 99% and 100% of the genomes for the 915 taxa tree and the 181 taxa tree, respectively. This yielded alignments of 2409 and 2026 amino acids, respectively. FastTree [56] was used to build the phylogeny using the LG + G substitution model (Fig. 1). The trees were plotted with the *ggtree* R package [57]. Pangenomic analysis of these 181 genomes was performed with anvi’o v8 [12, 58], which included functional annotation with the COG [59], KEGG [60], and CAZy [61] databases, ANI calculation with FastANI [50], computation of homologous gene clusters, and calculation of the geometric homogeneity index of each gene cluster [58] (Fig. 1). Geometric homogeneity compares the positions of gaps in the aligned residues without considering amino acid properties. To complement the ANI analysis, we also calculated the percent identity of full length 16S rRNA genes in the genomes, which were extracted with barrnap [62]. Estimated genome size was calculated by multiplying the assembly size by 100 and dividing by the checkM completeness percentage. Strains were classified as “symbiotic N-fixers” if they contained at least five of six key *nif* genes (*nifBDEHKN*) and at least four of five key nodulation genes (*nodABCIJ*) [31], “free-living N-fixers” if they only contained the *nif* genes and no *nod* genes, and “photosynthetic” if they contained the photosynthetic reaction center HML subunits. We also searched for a suite of genes involved in aerobic respiration, carbon fixation, denitrification, single carbon compound (carbon monoxide, methane, methanol) metabolism, and sulfur metabolism [63].

### Ecological analyses

The output of the Sylph profile analysis was the relative abundance of each *Bradyrhizobium* strain (relative only to the other *Bradyrhizobium* strains in the sample) in each of the 331 soil metagenomic samples. We used the *pheatmap* R package to plot this relative abundance matrix along with relevant metadata [64]. We calculated a weighted UniFrac distance matrix in the *phyloseq* R package [65, 66]. Weighted UniFrac distance was significantly positively correlated with Bray–Curtis dissimilarity (Mantel *r* = 0.23*, P* < .001), but we opted to use weighted UniFrac because the Bray–Curtis dissimilarity matrix had a skewed distribution with many “1” values due to lack of any shared taxa among many samples, whereas weighted UniFrac accounts for phylogenetic similarity in the distribution of the *Bradyrhizobium* strains across samples and offers a metric for comparing two samples with no overlapping strains. Weighted UniFrac distance was also significantly positively correlated with unweighted UniFrac distance (Mantel *r* = 0.6*, P* < .001). To include more predictor variables for certain analyses, we subset the data to 219 samples that had a suite of 13 soil chemistry variables available. We performed non-metric multidimensional scaling (NMDS) combined with the envfit function in the *vegan* R package [67] to visualize compositional differences and associations with environmental variables. We used distance-based redundancy analysis (dbRDA) and forward stepwise model selection in *vegan* to identify the top drivers of compositional differences. We partitioned variation attributed to geographic and dbRDA-selected environmental variables using the varpart function in *vegan*. We used partial Mantel tests in *vegan* to assess correlations between weighted UniFrac distances and geographic distance and environmental distance (Euclidean distance of dbRDA-selected variables). We also used generalized dissimilarity modeling in the *gdm* R package [68] to further assess the effects of geographic distance and environmental dissimilarity and assess their relative importance (Fig. 1). All plots besides the heatmap were made with the *ggplot2* R package [69]. All analyses were performed with R version 4.2.3 [70]. Downstream data and analyses are publicly available on Zenodo (DOI: http://dx.doi.org/10.5281/zenodo.15540063).

## Results

Based on 16S rRNA gene-based analyses of the metagenomes [41], *Bradyrhizobium* was found to be one of the most prevalent and abundant genera across the 331 Australian soils. *Bradyrhizobium* was detected in 307 of 331 samples, with relative abundances (compared to all other bacteria and archaea) ranging from 0% to 2.8%, with a mean of 0.5% and median of 0.4%.

To examine strain-level diversity of *Bradyrhizobium*, we then implemented a StrainFinder-based pipeline for the targeted assembly of *Bradyrhizobium* genomes from a subset of the Australian soil metagenomes. StrainFinder generated 212 *Bradyrhizobium* MAGs (four in each of 53 samples); we retained 106 high-quality *Bradyrhizobium* MAGs (the top two from each of 53 samples) for further analyses. Estimated completeness of those strains ranged from 98.8% to 100% and contamination ranged from 0% to 0.34% (Fig. S3). Average nucleotide identities (ANI) across the 106 strains ranged from 92 to 97%, and the ANI between the strains and the reference genome used in StrainFinder ranged from 93% to 97%. The percent identity of the full length 16S rRNA gene among the 106 StrainFinder MAGs ranged from 87.73% to 99.73%, and between the strains and the reference genome from 90.54% to 97.12%. All 106 StrainFinder MAGs were classified as genus *Bradyrhizobium* by GTDB-Tk, and the concatenated bac120 phylogenetic tree placed them in the *Bradyrhizobium* genus (Fig. S4).

**Figure 2:**
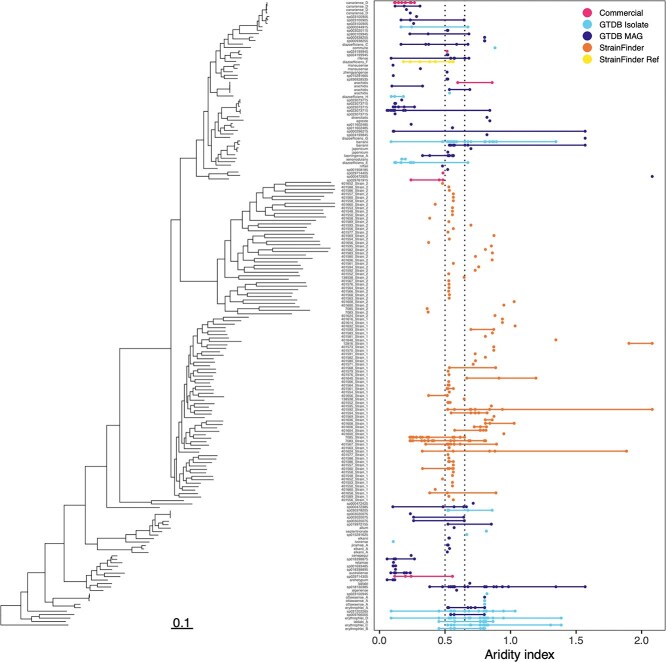
Presence of 181 *Bradyrhizobium* strains across the 268 of the soil metagenomes in which at least one *Bradyrhizobium* strain was detected by Sylph, ordered by aridity index. Dotted lines show the cutoffs for climate classes where arid to semi-arid is <0.5, dry sub-humid is 0.5 to 0.65, and humid is >0.65. For a similar figure showing relative abundance and other metadata, see Fig. S5. To the left is the phylogenetic tree of the 181 *Bradyrhizobium* strains based on a concatenated alignment of 2026 amino acids from the bac120 marker gene set (scalebar reflects substitutions per site). The tree is rooted with *Nitrobacter winogradsky* (not shown).

We next combined the 106 StrainFinder MAGs with 809 high-quality pre-existing *Bradyrhizobium* genomes from GTDB and used Sylph [46] to determine which *Bradyrhizobium* genomes were detected in each of the 331 soil metagenomes (Fig. S2). A total of 181 unique strains were detected (92 StrainFinder MAGs and 89 GTDB genomes) across 268 soils (81% of the soil metagenomes) (Fig. 2, Fig. S5). Of the 89 detected GTDB genomes, only 26 were from isolates, whereas a majority (63 genomes) were MAGs. The phylogeny of these 181 strains based on bac120 showed two clusters of GTDB genomes and a cluster of StrainFinder MAGs (Fig. 2). These 181 strains were distributed across the broader *Bradyrhizobium* phylogeny (Fig. S4). The strains detected included 6 of 19 commercial *Bradyrhizobium* strains [53]. All 53 of the most abundant StrainFinder MAGs were detected, whereas 39 of the 53 second most abundant StrainFinder MAGs were detected. The maximum number of strains detected per sample was 7, whereas the maximum number of samples a particular strain was detected in was 41. The estimated genome sizes for the 181 strains detected by Sylph ranged from ~7 to 12 MB. Prevalence (number of samples in which a given strain was detected) was not significantly associated with genome size (linear regression, *R*^2^ = 0.02*, P* = .06, Fig. S6) nor did genome size significantly differ between N-fixing and non-N-fixing *Bradyrhizobium* genomes (t-test, t = −0.66*, P* = .52). Patterns in the occurrence of strains in soils based on either aridity or mean annual temperature did not closely follow the phylogeny, but there was a high degree of variation in the range of temperatures the strains were detected in, with the widest range being 20.1°C (Fig. 2, Fig. S7). However, both aridity range and temperature range (for strains found in at least two soils) was more related to prevalence (linear regression, *R*^2^ = 0.34*, P* < .001; *R*^2^ = 0.30*, P* < .001, respectively) than to genome size (linear regression, *R*^2^ = 0.01*, P* = .52; *R*^2^ = 0.04*, P* = .08, respectively).

Among those 181 strains detected across the Australian soils, ANI ranged from 79.6% to 99.9% and full-length 16S rRNA gene percent identity (calculated for 179 strains that contained a full length 16S rRNA gene) ranged from 88% to 100%. Full length 16S rRNA gene percent identity among the most abundant StrainFinder MAGs and the GTDB genomes ranged from 92.76% to 100%, and among just the 89 detected GTDB genomes from 95.77 to 100%. These 181 strains would correspond to 82 operational taxonomic units (OTUs) at 99% similarity for the full-length 16S rRNA gene [71], 38 OTUs at 97% similarity for the V4 region of the 16 rRNA gene [72, 73], or 104 “species” at the 95% ANI cut-off [50, 74] (Fig. S8). ANI calculations were not affected by plasmid sequences; of the 181 genome assemblies, only two had assembled plasmids and these plasmids were < 1 MB in size.

**Figure 3:**
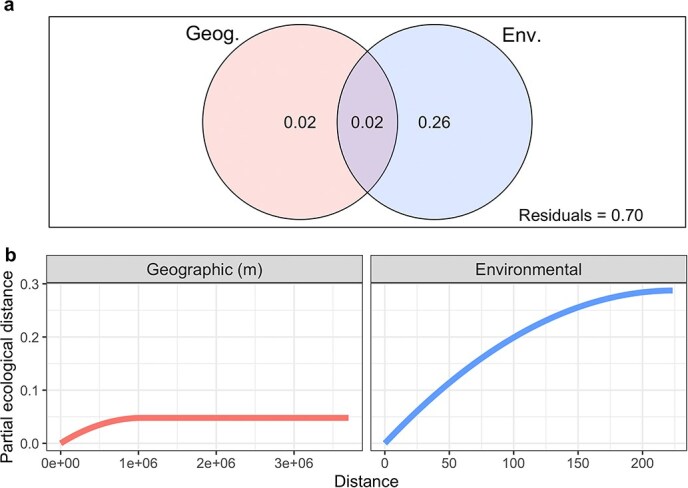
Relative importance of geography versus environment in structuring *Bradyrhizobium* strain-level community composition using two independent methods. (a) Variation partitioning of weighted UniFrac distance by geographic (latitude, longitude) and dbRDA-selected environmental variables (temperature, pH, manganese, aluminum, nitrate, and zinc). (b) Partial ecological distance splines from GDM of geographic (m) and environmental (Euclidean) distance. dbRDA and GDM were performed on a subset of 219 samples to include more environmental variables. See Table S3 for more statistical information, as well as corresponding partial Mantel test results.

*Bradyrhizobium* community composition, as determined by the relative abundances of the 181 strains in the 268 soil metagenomes (relative to total *Bradyrhizobium*), was significantly associated with several soil and climate variables Fig. S9, envfit*, P* = .001). Stepwise distance-based redundancy analysis (dbRDA) identified temperature, pH, manganese, aluminum, nitrate, and zinc as the most significant predictors of *Bradyrhizobium* community composition (*P* < .05, Table S2). Results were similar for both weighted and unweighted UniFrac metrics (Table S2). Variation partitioning among geographic distance and the dbRDA-selected environmental variables explained 30% of the variation in community composition, with environmental variables (26%) explaining much more than geographic distance (2%) (Table S3, Fig. 3a). A partial-Mantel test controlling for geographic distance found that community dissimilarity significantly increased with increasing environmental dissimilarity (*r* = 0.19*, P* < .001), whereas the opposite (testing the effect of geography while controlling for environment) was not true (*r* = −0.02, *P* = .75). Results were similar for both weighted and unweighted UniFrac metrics (Table S3). Generalized dissimilarity modeling found that, even though both spatial distance and environmental distance were significantly associated with both weighted and unweighted UniFrac distances (*P* < .05), environmental distance had greater maximum predicted ecological distance and variable importance scores than geographic distance (Table S3, Fig. 3b). In short, the biogeographical patterns observed for the *Bradyrhizobium* strains across the 268 soils were more closely associated with variation in environmental conditions (both climate and soil properties) than geographic distance per se.

**Figure 4:**
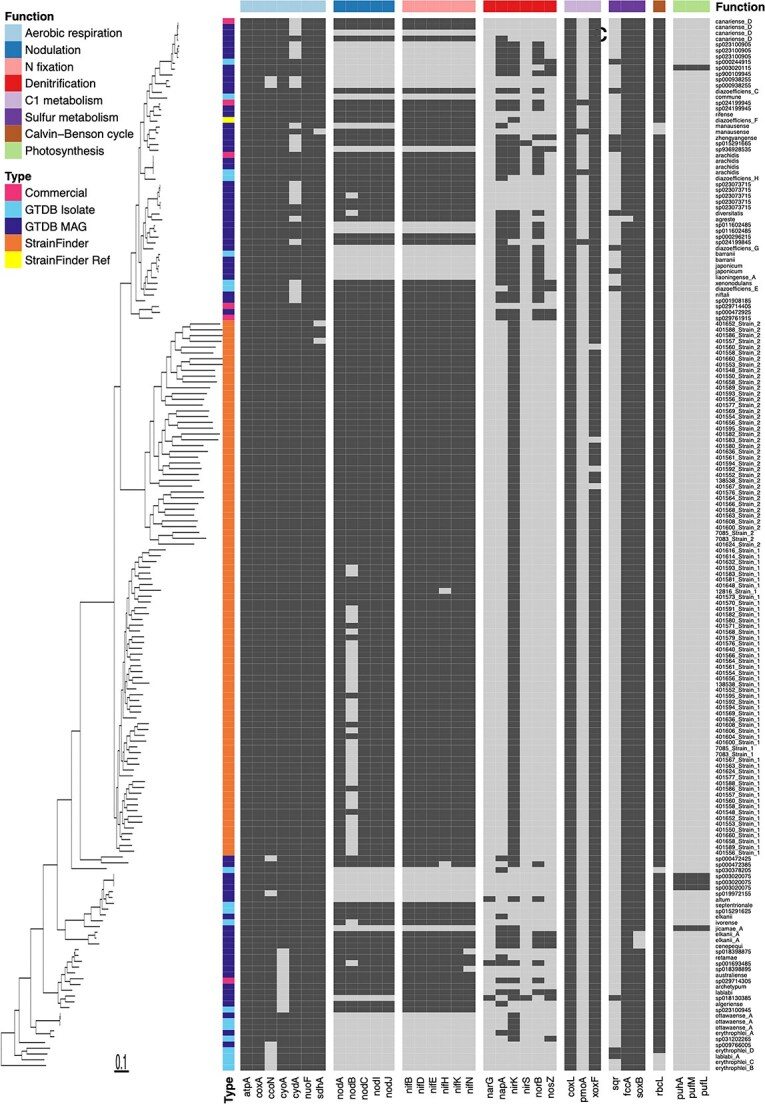
Presence (dark gray) or absence (light gray) of genes involved in aerobic respiration, nodulation, N-fixation, C1 metabolism (carbon monoxide, methane, and methanol), sulfur metabolism (sulfide and thiosulfate), the Calvin-Benson cycle, and photosynthesis, as well as genome type. The row order and phylogeny are the same as Fig. 2.

All 181 *Bradyrhizobium* strains detected contained genes for aerobic respiration (e.g. *atpA*, *coxA*) and carbon monoxide oxidation (*coxL*) (Fig. 4). Most strains also contained genes for methanol (*xoxF*), sulfide (*fccA*), and thiosulfate (*soxB*) oxidation, and the Calvin-Benson cycle (*rbcL*) for carbon fixation. There were also four stains containing genes for methane oxidation, 11 strains containing genes for the complete denitrification pathway, and 129 strains containing genes for partial denitrification. 145 strains contained *nifH* and other N-fixing genes, and 143 of those 145 also contained nodulation genes, indicating potential for symbiotic N-fixation. Only five strains contained photosynthesis genes, and those strains were not very prevalent, being detected in one to five samples (Fig. 2, Fig. 4).

There were 79 261 gene clusters identified in the pangenome out of 1 416 019 total genes; only a small proportion, 1935 (2%), were shared by all 181 strains, whereas a much larger number, 41 784 (53%), were present in only one strain (Fig. S10). However, the majority of gene clusters found in only one strain (73.56%) were of unknown function. Gene clusters that were present in more strains had lower geometric homogeneity than gene clusters that were present in only a few strains and this pattern was consistent across COG categories (Fig. S11). Gene clusters with the lowest geometric homogeneity (i.e. greatest variability) were in the “Mobilome: prophages, transposons” COG category, followed by the “Replication, recombination and repair” categories, both of which had values <0.85 across the gene clusters present in 10 to 172 strains (Fig. S11).

## Discussion

Here we describe a generalizable methodological framework for strain-based analyses of soil bacteria that we used to conduct a comprehensive assessment of the strain-level diversity and biogeography of a highly prevalent and abundant genus of soil bacteria. We built on previous work [25, 26] by examining strain-level diversity within a genus and across a large geographic area that spanned pronounced gradients in soil and climate conditions. Our results show that there is substantial strain-level diversity in *Bradyrhizobium* across soils of the Australian continent. Although we did not detect clear clustering of strains by aridity, in contrast to prior work on *Acacia*-associated *Bradyrhizobium* [8], strain-level community composition of bulk soil *Bradyrhizobium* across Australia was significantly associated with differences in environmental conditions, including mean annual temperature and soil characteristics such as soil pH and nitrate concentrations.

One reason to focus on *Bradyrhizobium*, in addition to its dominance in Australian soils, was the relatively large number of pre-existing reference genomes (compared to other soil genera). However, many of those genomes are from isolates that were cultured by growing plants in certain soils and then isolating the *Bradyrhizobium* from the nodules. Across Australian soils, we detected more MAGs (155) than isolate (26) genomes, highlighting the importance of cultivation-independent approaches for assessing the diversity of this genus in soil. We used StrainFinder to capture a broader diversity of *Bradyrhizobium* genomic diversity than is currently represented in the GTDB database. StrainFinder generated high-quality *Bradyrhizobium* MAGs based on the reference used, *B. diazoefficiens* F, which is part of the *B. japonicum* supergroup [35]. ANI values between the strains and the reference varied, meaning that StrainFinder successfully took into account the information contained in the individual soil metagenomes to generate new genomes based on the variation found in the sample, rather than just duplicating the reference genome. The StrainFinder MAGs were then detected independently by Sylph using the raw reads, including in the samples from which they were assembled, offering validation of this approach.

A total of 181 *Bradyrhizobium* strains were detected across the 331 soils, which is substantially more than the number of OTUs that would have been detected with 16S rRNA gene sequencing (Fig. S8) [6]. Additionally, we were able to obtain nearly complete genomes for all of the strains, making it possible to conduct more detailed genomic analyses and make ecological inferences. Furthermore, prior work has demonstrated that 16S rRNA gene sequences alone are often unable to classify members of the genus to the species level of resolution, another benefit of our genome-resolved approach [75]. A majority of the strains detected (80%) were N-fixers, which is contrary to the finding of a dominance of free-living *Bradyrhizobium* taxa in forests from across North America [37] and a lack of *nif* and *nod* genes in many other soil *Bradyrhizobium* strains [76]. Furthermore, we only detected two *Bradyrhizobium* strains that contained *nif* genes and lacked *nod* genes (i.e. free-living N-fixers), a phenomenon which has been reported in the literature [31, 77]; in our study, 143 of 145 strains with *nif* genes also contained *nod* genes, highlighting the prevalence of symbiotic N-fixers. There were no strains that contained *nod* genes but lacked *nif* genes, suggesting a lack of “cheaters” among these 181 strains [78]. The five photosynthetic strains did not contain *nod*, consistent with previous findings [79]. In addition to N-fixation and photosynthesis, we also detected *Bradyrhizobium* strains with genes for methane oxidation and both partial and complete denitrification [80–83]. Lastly, all 181 of the Australian *Bradyrhizobium* strains contained *coxL* for carbon monoxide oxidation [63, 84] and the *xoxF* gene for methanol oxidation, consistent with previous reports [85]. Together these results highlight the diversity of energy acquisition strategies across the 181 *Bradyrhizobium* strains we detected in the Australian soils.

Although agricultural soils were not included in the present study, we still detected the genomes of 6 out of 19 commercial *Bradyrhizobium* inoculants used in Australian agriculture [53], with one of them detected in 10 samples (Fig. 2). Whereas two (*B. canariense* D, *B. sp029714405*) of these six commercial inoculants were originally isolated from Australia, the other four were originally isolated from either South America (*B. sp024199945*, *B. sp029761915*, *B. sp029714305*) or Africa (*B. arachidis*). This raises the possibility that certain commercial agricultural inoculants have now spread into natural areas, a hypothesis in line with prior work showing that even foreign commercial inoculants have become naturalized in Australia over time [53].

The prevalence and relative abundances of *Bradyrhizobium* strains varied substantially across the Australian soils. The majority (98%) of strains were found in <10% of the soils (Fig. 2), but we acknowledge that individual strains could be more prevalent across soils and our inability to detect strains may be a product of rarity (and insufficient sequencing depth), not absence per se. Furthermore, despite the large and spatially extensive sampling, certain regions of the Australian continent were underrepresented in the samples used for this study. Notwithstanding these caveats, the strain-level biogeographic patterns observed across the genus were primarily associated with environmental dissimilarity rather than geographic distance. This result contrasts with previous evidence that *Bradyrhizobium* spp. can be dispersal limited [86].

*Bradyrhizobium* community composition was primarily associated with both soil properties and climate variables, namely temperature, pH, and concentrations of manganese, aluminum, nitrate, and zinc. Previous research on diazotrophs in forests, wheat fields, and alpine meadows found that the relative abundance of the genus *Bradyrhizobium* was positively associated with both temperature and precipitation and negatively associated with pH [32, 87, 88]. Our work shows that, while such a trend can be observed for the genus in aggregate, diverse temperature and pH preferences exist across different strains within the genus. Our results also suggest potential differences in aluminum and manganese tolerance among the 181 strains, which builds on previous work that found differences in aluminum and manganese tolerance among 16 *B. japonicum* strains [89]. Aridity was not a primary variable influencing *Bradyrhizobium* community composition, contrary to our expectation based on research on forest diazotrophs [32] and *Acacia*-associated *Bradyrhizobium japonicum* strains [8]. This could be due in part to the focus of our sampling on bulk soils from a wide range of vegetation types rather than a specific focus on forests or *Acacia*-associated *Bradyrhizobium* taxa. Alternatively, the biogeography of host-associated strains could be partly related to the aridity-associated differences in the distributions of host plant species.

Mean annual temperatures across the samples studied ranged from 6.7°C to 28.1°C and in the samples with *Bradyrhizobium*, from 7.6°C to 28.1°C, highlighting their presence across the majority of the Australian temperature gradient. Some strains had a broad temperature range whereas others had a narrow range, and temperature was the top predictor variable of weighted UniFrac distance. However, there were no broad divisions in the phylogeny with respect to low and high temperature preferences (Fig. S7). The importance of the soil and site variables is consistent with previous work on N-fixers which has shown that bacterial N-fixers as a whole functional group, including *Bradyrhizobium*, were structured more by soil properties than climate and vegetation in a Europe-wide survey [7]. In particular, that study found that relative abundances of N-fixing bacteria were greater in soils with lower pH. The importance of pH is in line with substantial previous work using amplicon sequencing of soil bacterial communities [90–92]; here we highlight, more specifically, that pH is also a key driver of strain-level variation in a highly prevalent and abundant genus.

Previous work on potential relationships between genomic traits and environmental distributions in *Bradyrhizobium* found that *Bradyrhizobium diazoefficiens* in more stressful environments (defined as environments with high temperature, low rainfall, high acidity, or high salinity) had smaller, more streamlined genomes [93]. In our study, across the 181 detected *Bradyrhizobium* strains, genome size was not significantly correlated with aridity, temperature, or pH. Future work could use a landscape genomic approach similar to that employed by Simonsen (2021) [93] to assess if other genomic traits besides genome size varied across those environmental gradients. An alternative hypothesis is that genome size could also be related to prevalence or the range of environmental conditions that an organism can tolerate [94]. Our results do not support this hypothesis either, as genome size was not significantly associated with ubiquity. Although it remains unclear why some strains are more prevalent than others and able to be detected in a larger number of soils that span a range in soil and site characteristics, the hypothesis that genome size is associated with ubiquity was not supported for members of this genus. However, it is worth noting that members of the *Bradyrhizobium* genus have larger genomes than other genera in the *Bradyrhizobiaceae* family, reflecting the general diversity in metabolisms and ecological strategies within the genus [36].

Given the broad spatial and environmental gradient examined here, as well as the genomic diversity encompassed by the 181 strains detected, we then asked which gene clusters in the pangenome were the most variable, in terms of presence/absence as well as geometric heterogeneity. The mobilome COG category was the most heterogeneous. However, the majority of gene clusters in each COG category were present in a low number (< 9) of strains (Fig. S11). The 181 strains comprised a large pangenome of 79 261 gene clusters, of which relatively few (< 3%) were in all strains, with many of the strains having unique gene clusters, similar to prior pangenomic analyses of *Bradyrhizobium* [76]. The high number of singleton gene clusters supports the idea of high genomic diversity in *Bradyrhizobium* across Australia. Previous genomic research on *Bradyrhizobium* suggested that *Bradyrhizobium* taxa contain modular systems consisting of many large integrative conjugative elements and few conjugative plasmids, which reshuffles genes and generates new combinations, which could contribute to the high number of singleton gene clusters in the pangenome [95].

## Conclusions

We sought to understand why a single bacterial genus is so prevalent and abundant in soil microbiomes by developing a general methodological framework for strain-level analyses of diverse microbial communities where pre-existing reference genome databases may not be sufficiently comprehensive (Fig. 1). Across broad environmental gradients at a continental spatial scale, our results demonstrate that there is a strain-level phylogenetic signal in community composition associated with key soil and climatic variables. Environmental dissimilarity explained more variation in *Bradyrhizobium* community composition than spatial distance. The Australian *Bradyrhizobium* pangenome was large and dominated by singleton gene clusters, with only <3% of gene clusters present in all 181 detected strains. The pangenome also demonstrated a breadth of metabolic strategies (Fig. 4) that is also likely key to the dominance of the genus. Future work should continue to investigate strain-level environmental distributions and genomic attributes of soil bacteria, particularly in dominant taxa relevant to agriculture and key ecosystem functions.

## Supplementary Material

Supplemental_Figures_wraf222

SupplementaryTables_wraf222

## Data Availability

Metagenomic sequences and sample metadata are available via the Bioplatforms Australia Data Portal (https://data.bioplatforms.com/bpa/otu/metagenome). The sample IDs and metadata downloaded from this platform are available in Table S1. Genome sequences for the 181 detected *Bradyrhizobium* genomes are available on figshare (https://dx.doi.org/10.6084/m9.figshare.29176142). Example terminal commands as well as downstream data and R analysis scripts are available on Zenodo (https://zenodo.org/records/ 17 211 138). The KBase narrative containing the StrainFinder work is publicly available with narrative ID 196529.

## References

[1] Thompson LR, Sanders JG, McDonald D. et al. A communal catalogue reveals Earth’s multiscale microbial diversity. *Nature* 2017;551:457–63. 10.1038/nature24621 PMC6192678 29088705

[2] Delgado-Baquerizo M, Oliverio AM, Brewer TE. et al. A global atlas of the dominant bacteria found in soil. *Science* 2018;359:320–5. 10.1126/science.aap9516 29348236

[3] Pedrós-Alió C . The rare bacterial biosphere. *Annu Rev Mar Sci* 2012;4:449–66. 10.1146/annurev-marine-120710-100948 22457983

[4] Bickel S, Or D. The chosen few—variations in common and rare soil bacteria across biomes. *The ISME Journal* 2021;15:3315–25. 10.1038/s41396-021-00981-3 34035442 PMC8528968

[5] Singleton CM. et al. Microflora Danica: the atlas of Danish environmental microbiomes. *bioRxiv* 2024. 10.1101/2024.06.27.600767

[6] Oliverio AM, Bissett A, McGuire K. et al. The role of phosphorus limitation in shaping soil bacterial communities and their metabolic capabilities. *mBio* 2020;11:e01718–20. 10.1128/mBio.01718-20 33109755 PMC7593963

[7] Labouyrie M, Ballabio C, Romero F. et al. Patterns in soil microbial diversity across Europe. *Nat Commun* 2023;14:3311. 10.1038/s41467-023-37937-4 PMC10250377 37291086

[8] Simonsen AK, Barrett LG, Thrall PH. et al. Novel model-based clustering reveals ecologically differentiated bacterial genomes across a large climate gradient. *Ecol Lett* 2019;22:2077–86. 10.1111/ele.13389 31612601

[9] Chanin RB, West PT, Wirbel J. et al. Intragenic DNA inversions expand bacterial coding capacity. *Nature* 2024;634:234–42. 10.1038/s41586-024-07970-4 39322669

[10] Cho J-C, Tiedje JM. Biogeography and degree of endemicity of fluorescent *pseudomonas* strains in soil. *Appl Environ Microbiol* 2000;66:5448–56. 10.1128/AEM.66.12.5448-5456.2000 PMC92480 11097926

[11] Coleman ML, Chisholm SW. Ecosystem-specific selection pressures revealed through comparative population genomics. *Proc Natl Acad Sci* 2010;107:18634–9. 10.1073/pnas.1009480107 20937887 PMC2972931

[12] Delmont TO, Eren AM. Linking pangenomes and metagenomes: the *Prochlorococcus* metapangenome. *PeerJ* 2018;6:e4320. 10.7717/peerj.4320 29423345 PMC5804319

[13] Olm MR, Dahan D, Carter MM. et al. Robust variation in infant gut microbiome assembly across a spectrum of lifestyles. *Science* 2022;376:1220–3. 10.1126/science.abj2972 PMC9894631 35679413

[14] Chen L, Wang D, Garmaeva S. et al. The long-term genetic stability and individual specificity of the human gut microbiome. *Cell* 2021;184:2302–2315.e12. 10.1016/j.cell.2021.03.024 33838112

[15] Segata N . On the road to strain-resolved comparative metagenomics. *mSystems* 2018;3:e00190–17. 10.1128/mSystems.00190-17 29556534 PMC5850074

[16] Van Rossum T. et al. Diversity within species: interpreting strains in microbiomes. *Nat Rev Microbiol* 2020;18:491–506. 10.1038/s41579-020-0368-1 32499497 PMC7610499

[17] Anyansi C, Straub TJ, Manson AL. et al. Computational methods for strain-level microbial detection in Colony and metagenome sequencing data. *Front Microbiol* 2020;11:11. 10.3389/fmicb.2020.01925 PMC7507117 33013732

[18] Ventolero MF, Wang S, Hu H. et al. Computational analyses of bacterial strains from shotgun reads. *Brief Bioinform* 2022;23:bbac013. 10.1093/bib/bbac013 35136954

[19] Ghazi AR, Münch PC, Chen D. et al. Strain identification and quantitative analysis in microbial communities. *J Mol Biol* 2022;434:167582. 10.1016/j.jmb.2022.167582 35398320

[20] Ma S, Li H. Statistical and computational methods for microbial strain analysis. In: Fridley B, Wang X (eds), Statistical Genomics. New York, NY: Springer US, 2023, 231–45, New York, NY, 10.1007/978-1-0716-2986-4_11. 36929080

[21] Olm MR, Crits-Christoph A, Bouma-Gregson K. et al. inStrain profiles population microdiversity from metagenomic data and sensitively detects shared microbial strains. *Nat Biotechnol* 2021;39:727–36. 10.1038/s41587-020-00797-0 33462508 PMC9223867

[22] Wang S, Jiang Y, Li S. PStrain: an iterative microbial strains profiling algorithm for shotgun metagenomic sequencing data. *Bioinformatics* 2021;36:5499–506. 10.1093/bioinformatics/btaa1056 33346799

[23] Smillie CS, Sauk J, Gevers D. et al. Strain tracking reveals the determinants of bacterial engraftment in the human gut following Fecal microbiota transplantation. *Cell Host Microbe* 2018;23:229–240.e5. 10.1016/j.chom.2018.01.003 29447696 PMC8318347

[24] Zhang Z, Wang J, Wang J. et al. Estimate of the sequenced proportion of the global prokaryotic genome. *Microbiome* 2020;8:134. 10.1186/s40168-020-00903-z 32938501 PMC7496214

[25] Crits-Christoph A, Olm MR, Diamond S. et al. Soil bacterial populations are shaped by recombination and gene-specific selection across a grassland meadow. *The ISME Journal* 2020;14:1834–46. 10.1038/s41396-020-0655-x 32327732 PMC7305173

[26] Sjöqvist C, Delgado LF, Alneberg J. et al. Ecologically coherent population structure of uncultivated bacterioplankton. *The ISME Journal* 2021;15:3034–49. 10.1038/s41396-021-00985-z 33953362 PMC8443644

[27] Van Rossum T. et al. metaSNV v2: detection of SNVs and subspecies in prokaryotic metagenomes. *Bioinformatics* 2022;38:1162–4. 10.1093/bioinformatics/btab789 34791031 PMC8796361

[28] Xu J, Zhang Y, Zhang P. et al. The structure and function of the global citrus rhizosphere microbiome. *Nat Commun* 2018;9:4894. 10.1038/s41467-018-07343-2 PMC6244077 30459421

[29] Panzieri M, Marchettini N, Hallam TG. Importance of the *Bradhyrizobium japonicum* symbiosis for the sustainability of a soybean cultivation. *Ecol Model* 2000;135:301–10. 10.1016/S0304-3800(00)00383-5

[30] Antoun H, Beauchamp CJ, Goussard N. et al. Potential of *rhizobium* and *Bradyrhizobium* species as plant growth promoting rhizobacteria on non-legumes: effect on radishes (*Raphanus sativus* L.). *Plant Soil* 1998;204:57–67. 10.1023/A:1004326910584

[31] Tao J, Wang S, Liao T. et al. Evolutionary origin and ecological implication of a unique nif island in free-living *Bradyrhizobium* lineages. *The ISME Journal* 2021;15:3195–206. 10.1038/s41396-021-01002-z 33990706 PMC8528876

[32] Zhao W, Kou Y, Wang X. et al. Broad-scale distribution of diazotrophic communities is driven more by aridity index and temperature than by soil properties across various forests. *Glob Ecol Biogeogr* 2020;29:2119–30. 10.1111/geb.13178

[33] Han L-L, Wang Q, Shen JP. et al. Multiple factors drive the abundance and diversity of the diazotrophic community in typical farmland soils of China. *FEMS Microbiol Ecol* 2019;95:fiz113. 10.1093/femsec/fiz113 31295349

[34] Meng H, Zhou Z, Wu R. et al. Diazotrophic microbial community and abundance in acidic subtropical natural and re-vegetated forest soils revealed by high-throughput sequencing of nifH gene. *Appl Microbiol Biotechnol* 2019;103:995–1005. 10.1007/s00253-018-9466-7 30474727

[35] Avontuur JR, Palmer M, Beukes CW. et al. Genome-informed *Bradyrhizobium* taxonomy: where to from here? *Syst Appl Microbiol* 2019;42:427–39. 10.1016/j.syapm.2019.03.006 31031014

[36] Ormeño-Orrillo E, Martínez-Romero E. A Genomotaxonomy view of the *Bradyrhizobium* genus. *Front Microbiol* 2019;10:10. 10.3389/fmicb.2019.01334 31263459 PMC6585233

[37] VanInsberghe D, Maas KR, Cardenas E. et al. Non-symbiotic *Bradyrhizobium* ecotypes dominate north American forest soils. *The ISME Journal* 2015;9:2435–41. 10.1038/ismej.2015.54 25909973 PMC4611507

[38] Bissett A, Fitzgerald A, Court L. et al. Introducing BASE: the biomes of Australian soil environments soil microbial diversity database. *GigaSci* 2016;5:21. 10.1186/s13742-016-0126-5 PMC4870752 27195106

[39] Zomer RJ, Xu J, Trabucco A. Version 3 of the global aridity index and potential evapotranspiration database. *Sci Data* 2022;9:409. 10.1038/s41597-022-01493-1 35840601 PMC9287331

[40] Fick SE, Hijmans RJ. WorldClim 2: new 1-km spatial resolution climate surfaces for global land areas. *Int J Climatol* 2017;37:4302–15. 10.1002/joc.5086

[41] Salazar G, Ruscheweyh HJ, Hildebrand F. et al. mTAGs: taxonomic profiling using degenerate consensus reference sequences of ribosomal RNA genes. *Bioinformatics* 2021;38:270–2. 10.1093/bioinformatics/btab465 34260698 PMC8696115

[42] Quast C, Pruesse E, Yilmaz P. et al. The SILVA ribosomal RNA gene database project: improved data processing and web-based tools. *Nucleic Acids Res* 2013;41:D590–6. 10.1093/nar/gks1219 23193283 PMC3531112

[43] Rognes T, Flouri T, Nichols B. et al. VSEARCH: a versatile open source tool for metagenomics. *PeerJ* 2016;4:e2584. 10.7717/peerj.2584 PMC5075697 27781170

[44] Parks DH, Chuvochina M, Rinke C. et al. GTDB: an ongoing census of bacterial and archaeal diversity through a phylogenetically consistent, rank normalized and complete genome-based taxonomy. *Nucleic Acids Res* 2022;50:D785–94. 10.1093/nar/gkab776 34520557 PMC8728215

[45] Cox E, Tsuchiya MTN, Ciufo S. et al. NCBI taxonomy: enhanced access via NCBI datasets. *Nucleic Acids Res* 2025;53:D1711–5. 10.1093/nar/gkae967 39470745 PMC11701650

[46] Shaw J, Yu YW. Rapid species-level metagenome profiling and containment estimation with sylph. *Nat Biotechnol* 2024;43:1348–59. 10.1038/s41587-024-02412-y PMC12339375 39379646

[47] Aroney STN, Newell RJP, Nissen JN. et al. CoverM: read alignment statistics for metagenomics. *Bioinformatics* 2025;41:btaf147. 10.1093/bioinformatics/btaf147 40193404 PMC11993303

[48] Grönemeyer JL, Reinhold-Hurek B. Diversity of Bradyrhizobia in Subsahara Africa: a rich resource. *Front Microbiol* 2018;9:9. 10.3389/fmicb.2018.02194 30294308 PMC6158577

[49] Arkin AP, Cottingham RW, Henry CS. et al. KBase: the United States Department of Energy Systems Biology Knowledgebase. *Nat Biotechnol* 2018;36:566–9. 10.1038/nbt.4163 PMC6870991 29979655

[50] Jain C, Rodriguez-R LM, Phillippy AM. et al. High throughput ANI analysis of 90K prokaryotic genomes reveals clear species boundaries. *Nat Commun* 2018;9:5114. 10.1038/s41467-018-07641-9 PMC6269478 30504855

[51] Parks DH, Imelfort M, Skennerton CT. et al. CheckM: assessing the quality of microbial genomes recovered from isolates, single cells, and metagenomes. *Genome Res* 2015;25:1043–55. 10.1101/gr.186072.114 25977477 PMC4484387

[52] Chaumeil P-A, Mussig AJ, Hugenholtz P. et al. GTDB-Tk v2: memory friendly classification with the genome taxonomy database. *Bioinformatics* 2022;38:5315–6. 10.1093/bioinformatics/btac672 PMC9710552 36218463

[53] Kohlmeier MG, O'Hara GW, Ramsay JP. et al. Closed genomes of commercial inoculant rhizobia provide a blueprint for management of legume inoculation. *Appl Environ Microbiol* 2025;91:e0221324–4. 10.1128/aem.02213-24 39791879 PMC11837538

[54] Liao H, Ji Y, Sun Y. High-resolution strain-level microbiome composition analysis from short reads. *Microbiome* 2023;11:183. 10.1186/s40168-023-01615-w PMC10433603 37587527

[55] Starkenburg SR, Chain PSG, Sayavedra-Soto LA. et al. Genome sequence of the chemolithoautotrophic nitrite-oxidizing bacterium *Nitrobacter winogradskyi* Nb-255. *Appl Environ Microbiol* 2006;72:2050–63. 10.1128/AEM.72.3.2050-2063.2006 PMC1393235 16517654

[56] Price MN, Dehal PS, Arkin AP. FastTree: computing large minimum evolution trees with profiles instead of a distance matrix. *Mol Biol Evol* 2009;26:1641–50. 10.1093/molbev/msp077 19377059 PMC2693737

[57] Yu G, Smith DK, Zhu H. et al. ggtree: an r package for visualization and annotation of phylogenetic trees with their covariates and other associated data. *Methods Ecol Evol* 2017;8:28–36. 10.1111/2041-210X.12628

[58] Eren AM, Esen ÖC, Quince C. et al. Anvi’o: an advanced analysis and visualization platform for ‘omics data. *PeerJ* 2015;3:e1319. 10.7717/peerj.1319 26500826 PMC4614810

[59] Galperin MY, Makarova KS, Wolf YI. et al. Expanded microbial genome coverage and improved protein family annotation in the COG database. *Nucleic Acids Res* 2015;43:D261–9. 10.1093/nar/gku1223 25428365 PMC4383993

[60] Aramaki T, Blanc-Mathieu R, Endo H. et al. KofamKOALA: KEGG ortholog assignment based on profile HMM and adaptive score threshold. *Bioinformatics* 2020;36:2251–2. 10.1093/bioinformatics/btz859 31742321 PMC7141845

[61] Drula E, Garron ML, Dogan S. et al. The carbohydrate-active enzyme database: functions and literature. *Nucleic Acids Res* 2022;50:D571–7. 10.1093/nar/gkab1045 PMC8728194 34850161

[62] Seeman T. barrnap. 2018. https://github.com/tseemann/barrnap.

[63] Bay SK, Dong X, Bradley JA. et al. Trace gas oxidizers are widespread and active members of soil microbial communities. *Nat Microbiol* 2021;6:246–56. 10.1038/s41564-020-00811-w 33398096

[64] Kolde R . pheatmap: Pretty Heatmaps. R Package Version 1.0.12. https://CRAN.R-project.org/package=pheatmap, 2019.

[65] Lozupone C, Lladser ME, Knights D. et al. UniFrac: an effective distance metric for microbial community comparison. *ISME J* 2011;5:169–72. 10.1038/ismej.2010.133 20827291 PMC3105689

[66] McMurdie PJ, Holmes S. phyloseq: an R package for reproducible interactive analysis and graphics of microbiome census data. *PLoS One* 2013;8:e61217. 10.1371/journal.pone.0061217 PMC3632530 23630581

[67] Oksanen J. et al. vegan: Community Ecology Package. R Package Version 2.5–6. https://CRAN.R-project.org/package=vegan. 2022.

[68] Fitzpatrick M. et al. gdm: Generalized Dissimilarity Modeling. R Package Version 1.5.0–9.1. https://CRAN.R-project.org/package=gdm. 2022.

[69] Wickham H. ggplot2: Elegant Graphics for Data Analysis. New York, NY: Springer-Verlag, 2016, 10.1007/978-3-319-24277-4.

[70] R Core Team . R: A Language and Environment for Statistical Computing, https://www.R-project.org/. (2023, date last accessed).

[71] Edgar RC . Updating the 97% identity threshold for 16S ribosomal RNA OTUs. *Bioinformatics* 2018;34:2371–5. 10.1093/bioinformatics/bty113 29506021

[72] DeSantis TZ, Hugenholtz P, Larsen N. et al. Greengenes, a chimera-checked 16S rRNA gene database and workbench compatible with ARB. *Appl Environ Microbiol* 2006;72:5069–72. 10.1128/AEM.03006-05 16820507 PMC1489311

[73] Caporaso JG, Kuczynski J, Stombaugh J. et al. QIIME allows analysis of high-throughput community sequencing data. *Nat Methods* 2010;7:335–6. 10.1038/nmeth.f.303 20383131 PMC3156573

[74] Olm MR, Crits-Christoph A, Diamond S. et al. Consistent metagenome-derived metrics Verify and Delineate bacterial species boundaries. *mSystems* 2020;5. 10.1128/msystems.00731-19 PMC6967389 31937678

[75] Willems A, Coopman R, Gillis M. Phylogenetic and DNA-DNA hybridization analyses of *Bradyrhizobium* species. *Int J Syst Evol Microbiol* 2001;51:111–7. 10.1099/00207713-51-1-111 11211247

[76] Zhong C, Hu G, Hu C. et al. Comparative genomics analysis reveals genetic characteristics and nitrogen fixation profile of *Bradyrhizobium*. *iScience* 2024;27:108948. 10.1016/j.isci.2024.108948 PMC10845061 38322985

[77] Patra D, Mandal S. Nod–factors are dispensable for nodulation: a twist in bradyrhizobia-legume symbiosis. *Symbiosis* 2022;86:1–15. 10.1007/s13199-021-00826-9

[78] Sachs JL, Ehinger MO, Simms EL. Origins of cheating and loss of symbiosis in wild *Bradyrhizobium*. *J Evol Biol* 2010;23:1075–89. 10.1111/j.1420-9101.2010.01980.x 20345811

[79] Giraud E, Moulin L, Vallenet D. et al. Legumes symbioses: absence of nod genes in photosynthetic Bradyrhizobia. *Science* 2007;316:1307–12. 10.1126/science.1139548 17540897

[80] Shan J, Sanford RA, Chee-Sanford J. et al. Beyond denitrification: the role of microbial diversity in controlling nitrous oxide reduction and soil nitrous oxide emissions. *Glob Chang Biol* 2021;27:2669–83. 10.1111/gcb.15545 33547715

[81] Liu D, Yang Y, Ai J. et al. Research on microbial structures, functions and metabolic pathways in an advanced denitrification system coupled with aerobic methane oxidation based on metagenomics. *Bioresour Technol* 2021;332:125047. 10.1016/j.biortech.2021.125047 33839509

[82] Minamisawa K . Mitigation of greenhouse gas emission by nitrogen-fixing bacteria. *Biosci Biotechnol Biochem* 2023;87:7–12. 10.1093/bbb/zbac177 36354103

[83] Meng H, Wu R, Wang YF. et al. A comparison of denitrifying bacterial community structures and abundance in acidic soils between natural forest and re-vegetated forest of Nanling nature reserve in southern China. *J Environ Manag* 2017;198:41–9. 10.1016/j.jenvman.2017.04.066 28500915

[84] Lorite MJ, Tachil J̈, Sanjuán J́. et al. Carbon monoxide dehydrogenase activity in *Bradyrhizobium japonicum*. *Appl Environ Microbiol* 2000;66:1871–6. 10.1128/AEM.66.5.1871-1876.2000 PMC101426 10788353

[85] Bao Z, Okubo T, Kubota K. et al. Metaproteomic identification of diazotrophic methanotrophs and their localization in root tissues of field-grown Rice plants. *Appl Environ Microbiol* 2014;80:5043–52. 10.1128/AEM.00969-14 PMC4135783 24928870

[86] Bissett A. et al. Life history determines biogeographical patterns of soil bacterial communities over multiple spatial scales. *Mol Ecol* 2010;19:4315–27. 10.1111/j.1365-294X.2010.04804.x 25241408

[87] Wang Y, Li C, Kou Y. et al. Soil pH is a major driver of soil diazotrophic community assembly in Qinghai-Tibet alpine meadows. *Soil Biol Biochem* 2017;115:547–55. 10.1016/j.soilbio.2017.09.024

[88] Fan K, Weisenhorn P, Gilbert JA. et al. Soil pH correlates with the co-occurrence and assemblage process of diazotrophic communities in rhizosphere and bulk soils of wheat fields. *Soil Biol Biochem* 2018;121:185–92. 10.1016/j.soilbio.2018.03.017

[89] Indrasumunar A, Menzies NW, Dart PJ. Laboratory prescreening of *Bradyrhizobium japonicum* for low pH, Al and Mn tolerance can be used to predict their survival in acid soils. *Soil Biol Biochem* 2012;48:135–41. 10.1016/j.soilbio.2012.01.019

[90] Lauber CL, Hamady M, Knight R. et al. Pyrosequencing-based assessment of soil pH as a predictor of soil bacterial community structure at the continental scale. *Appl Environ Microbiol* 2009;75:5111–20. 10.1128/AEM.00335-09 PMC2725504 19502440

[91] Lammel DR, Barth G, Ovaskainen O. et al. Direct and indirect effects of a pH gradient bring insights into the mechanisms driving prokaryotic community structures. *Microbiome* 2018;6:106. 10.1186/s40168-018-0482-8 PMC5996553 29891000

[92] Zhalnina K, Dias R, de Quadros PD. et al. Soil pH determines microbial diversity and composition in the park grass experiment. *Microb Ecol* 2015;69:395–406. 10.1007/s00248-014-0530-2 25395291

[93] Simonsen AK . Environmental stress leads to genome streamlining in a widely distributed species of soil bacteria. *The ISME Journal* 2022;16:423–34. 10.1038/s41396-021-01082-x 34408268 PMC8776746

[94] Barberán A, Ramirez KS, Leff JW. et al. Why are some microbes more ubiquitous than others? Predicting the habitat breadth of soil bacteria. *Ecol Lett* 2014;17:794–802. 10.1111/ele.12282 24751288

[95] Weisberg AJ, Rahman A, Backus D. et al. Pangenome evolution reconciles robustness and instability of Rhizobial Symbiosis. *mBio* 2022;13:e0007422–2. 10.1128/mbio.00074-22 35416699 PMC9239051

